# Comment on “Effectiveness of Physiotherapy for Ventilator-Associated Pneumonia”

**DOI:** 10.1155/2012/329843

**Published:** 2012-07-02

**Authors:** George Ntoumenopoulos

**Affiliations:** Physiotherapy Department, Guy's and St Thomas' NHS Foundation Trust, Kings Health Partners, London SE1 7EH, UK

Dear Editor 

I read with interest the review from Hellweg [1]. However the findings of Ntoumenopoulos et al. [2] were in-accurately reported. 

Ntoumenopoulos et al. [2] investigated the effect of chest physiotherapy in sixty adult patients intubated and mechanically ventilated for at least 48-hours. There were no differences in the duration of mechanical ventilation, length of stay in ICU or mortality. Ventilator-associated pneumonia (VAP) as assessed by combined clinical assessment and the clinical pulmonary infection score (CPIS) occurred in 39% of the control group and 8% of the intervention group (*P* = 0.02). After adjustment was made by logistic regression for other important variables, chest physiotherapy was independently associated with a reduced occurrence of VAP (*P* = 0.02). Therefore, contrary to the conclusions by Hellwegg [1] the work of Ntoumenopoulos et al. [2] provides preliminary evidence that chest physiotherapy in ventilated patients was independently associated with a reduction in VAP. 

A randomised controlled trial by Pattanshetty and Gaude [3] reported that twice-daily chest physiotherapy was associated with a significant decrease in the clinical pulmonary infection scores (surrogate measure of VAP) and with a significant reduction in mortality. Blot et al. [4] therefore recommend the further investigation of chest physiotherapy for the prevention of VAP.
